# PTEN Protein Loss and Loss-of-Function Mutations in Gastric Cancers: The Relationship with Microsatellite Instability, EBV, HER2, and PD-L1 Expression

**DOI:** 10.3390/cancers12071724

**Published:** 2020-06-29

**Authors:** Binnari Kim, So Young Kang, Deokgeun Kim, You Jeong Heo, Kyoung-Mee Kim

**Affiliations:** 1 Department of Pathology and Translational Genomics, Samsung Medical Center, Sungkyunkwan University School of Medicine, Seoul 06351, Korea; 0734953@uuh.ulsan.kr (B.K.); sy500.kang@samsung.com (S.Y.K.); 2 Center of Companion Diagnostics, Samsung Medical Center, Seoul 06351, Korea; 3 Department of Pathology, Ulsan University Hospital, University of Ulsan College of Medicine, Ulsan 44033, Korea; 4 Department of Clinical Genomics, Samsung Medical Center, Seoul 06351, Korea; deokgeum.kim@samsung.com; 5 The Samsung Advanced Institute for Health Sciences & Technology (SAIHST), Samsung Medical Center, Sungkyunkwan University School of Medicine, Seoul 06351, Korea; heo893@naver.com

**Keywords:** PTEN, nonsense mutation, inactivation, gastric cancer

## Abstract

Inactivation of phosphatase and tensin homolog (PTEN) is caused by multiple mechanisms, and loss of PTEN activity is related to the progression of various cancers. In gastric cancer (GC), the relationship between the loss of PTEN protein expression and various genetic alterations remains unclear. The effects of microsatellite instability (MSI), Epstein–Barr virus (EBV), HER2 overexpression, and PD-L1 expression on *PTEN* mutation have not been fully explored. We performed comprehensive cancer panel tests with a cohort of 322 tumor samples from patients with advanced GC. Immunohistochemistry for PTEN protein was performed in all cases, and the loss of protein expression was defined as a complete absence of nuclear staining. In total, 34 cases (10.6%) had pathogenic *PTEN* mutations, of which 19 (55.9%) showed PTEN protein loss. The most common *PTEN* variants associated with protein loss were p.R130 (*n* = 4) followed by p.R335, p.L265fs, and deletions (*n* = 2). All the ten nonsense mutations identified in the samples resulted in PTEN inactivation. In the remaining 288 GC cases with wild-type *PTEN*, protein loss was found in 35 cases (12.2%). Thus, *PTEN* mutations were significantly associated with PTEN protein loss (*p* = 5.232 × 10^−10^), high MSI (*p* = 3.936 × 10^−8^), and EBV-positivity (*p* = 0.0071). In conclusion, our results demonstrate that loss-of-function mutations in *PTEN* are a frequent genetic mechanism of PTEN inactivation in GC.

## 1. Introduction

Mechanisms of DNA damage repair are crucial to preserve the genomic integrity of the cell. In normal cells, DNA damage triggers the activation of DNA damage response (DDR) pathways to repair the damage, and these depend on the type of DNA damage. The phosphatase and tensin homolog (*PTEN*), a well-known tumor suppressor gene, is involved in double-strand break repair, and nucleotide excision repair, and regulates the DDR pathways by interacting with Chk1 and p53 [1,2]. In addition, PTEN is a dual-specificity protein and lipid phosphatase that regulates various cellular processes and signal pathways, such as the induction of apoptosis by inhibiting phosphatidylinositol 3-kinase (PI3K)/Akt pathway and control of cell adhesion, migration, and tumor invasion, by downregulating the activity of focal adhesion kinases (FAKs) [3].

Somatic *PTEN* mutation, a frequent event in endometrial cancer, is associated with microsatellite instability (MSI) status ranging from 25% to 83% [4], and PTEN inactivation is thought to be an early step in the development and progression of endometrial cancer [5]. PTEN inactivation is also frequently observed in glioblastoma, with hemizygous or homozygous deletions in over 90% of primary glioblastomas [6]. In gastric cancers (GCs), the frequency of *PTEN* mutation is relatively low (7–11%) and mostly found in advanced GCs [7,8]. PTEN inactivation is closely linked to disease progression in GC. A gradual decrease in PTEN expression has been reported during the course of GC development from normal gastric mucosae, atrophic gastritis, intestinal metaplasia, dysplasia, and early stage GC to advanced stage GC [9,10]. Low PTEN expression is also associated with lymph node metastasis, advanced stage, diffuse type histology, and poor prognosis [11,12,13]. Functional inactivation of the tumor suppressor protein PTEN has been detected in multiple cases of GC, and already shown to be closely linked to the development, progression, and prognosis of the disease [3].

PTEN inactivation is known to be caused by various mechanisms in GC, including gene mutations, loss of heterozygosity, promoter hypermethylation, miRNA-mediated regulation, and post-translational phosphorylation [3]. Previous studies on *PTEN* mutation in GC showed no consensus on the frequency and type of *PTEN* mutation, because they were based on a small number of GC cases in various stages, and used direct sequencing or polymerase chain reaction single strand conformation polymorphism (PCR-SSCP) to detect the *PTEN* mutations, which also detected nonpathogenic mutations [14,15,16,17,18]. The relationship between PTEN protein loss and genetic variants remains unclear, and their effect on MSI, EBV, and PD-L1 expression has not yet been studied. Therefore, it is necessary to understand the association between *PTEN* mutations and its expression and other biomarkers of immunotherapy in advanced GC using a large cohort and a new detection method that can cover non-hot-spot mutations and exclude nonpathogenic mutations.

In this study, we performed next generation sequencing (NGS) in 322 patients with advanced GC and evaluated the association of *PTEN* mutation with its MSI, EBV, and PD-L1 expressions to evaluate its clinical significance in GC.

## 2. Results

### 2.1. Clinicopathological Characteristics of Patients with PTEN-Mutated GC

Of the 322 GC cases with NGS data included in this study, 38 showed pathogenic *PTEN* alterations confirmed by COSMIC [19], Polyphen [20], and SIFT [21]. Among them, three cases with low sequencing depth and one with poor quality were excluded, and finally, 34 cases (10.6%) were confirmed to harbor pathogenic *PTEN* mutations.

In the 34 GC cases with *PTEN* mutations, the median age of the patients was 64 years (40–85 years), and 22 (64.7%) patients were male. All the samples used for NGS analysis were from the stomach, of which 20 (58.8%) were resection specimens. In the pathologic subtypes, 31 cases (91.2%) were tubular adenocarcinoma, two (5.9%) were papillary adenocarcinoma, and one (2.9%) was a signet ring cell carcinoma. Eight cases (23.5%) were MSI-high, two (5.9%) were EBV-positive, and 24 (70.6%) were PD-L1 positive (CPS ≥ 1) among the 33 available cases. Five (14.7%) of the 34 cases were HER2-positive. The clinicopathologic characteristics of *PTEN*-mutated GCs are summarized in Table 1.

### 2.2. Clinicopathologic Characteristics of PTEN Wild-Type GC

In the GCs without *PTEN* alteration, the median age of the patients was 61 years (29–85 years), and 181 (62.8%) patients were male. All the samples used for NGS analysis were resected stomach specimens. Nine (3.1%) cases were MSI-high, 39 cases (13.5%) were HER2-positive, and seven (2.4%) cases were EBV-positive. PD-L1 positivity (CPS ≥ 1) was present in 182 (63.2%) out of the 287 available cases. Clinicopathologic characteristics of *PTEN* wild-type GCs are summarized in Table S1.

### 2.3. PTEN Mutations in Relationship with PTEN Protein Loss, MSI, EBV, and PD-L1 Expression 

**Figure 1:**
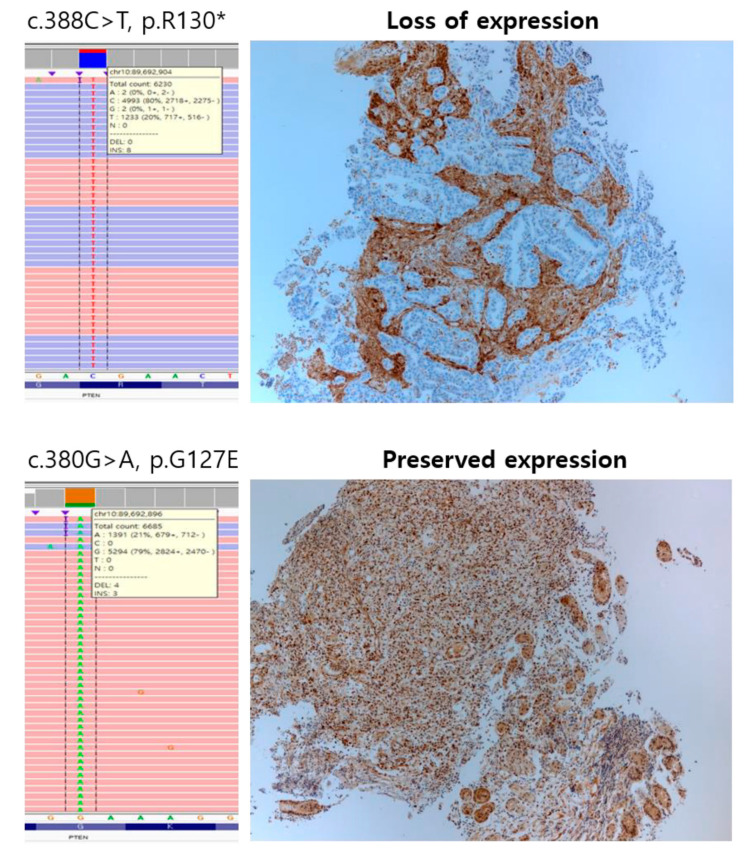
Representative cases of Phosphatase and Tensin Homolog (PTEN) immunohistochemistry and *PTEN* mutation confirmed by Integrative Genomics Viewer.

In the 34 *PTEN*-mutated GCs, we detected a total of 38 *PTEN* alterations of which four cases had two variants each. *PTEN* alterations were composed of 18 missense mutations, ten nonsense mutations, seven frameshift mutations, two deletions, and one nonframeshift deletion. The most frequent *PTEN* mutation was p.R130 (4/38, 10.5%), followed by p.L265fs (3/38, 7.9%). Among them, more than half of the cases showed PTEN protein loss (19/34, 55.9%), which was found in ten nonsense mutations, four missense mutations, three frameshift mutations, two deletions, and one non-frameshift deletion of *PTEN*, and one case with PTEN inactivation had two nonsense mutations. All cases with nonsense mutation showed PTEN protein loss. The most common *PTEN* mutation associated with protein loss was p.R130 (*n* = 4), followed by p.R335, p.L265fs, and deletion (*n* = 2). Representative cases of PTEN IHC and *PTEN* mutation confirmed by Integrative Genomics Viewer are shown in Figure 1. *PTEN* mutation was significantly associated with PTEN protein loss (*p* = 5.232 × 10^−10^), MSI-high (*p* = 3.936 × 10^−8^), and EBV-positivity (*p* = 0.0071) but was not associated with sex (*p* = 0.9805), age (*p* = 0.175), HER2 status (*p* = 0.79), and PD-L1 CPS (*p* = 0.111). 

Comparison of the clinicopathologic characteristics of *PTEN*-mutated and *PTEN* wild-type GCs are summarized in Table 2. PTEN inactivation was significantly associated with disease stages (*p* = 0.04), but did not predict overall survival (*p* = 0.083).

### 2.4. Co-Occurring Genetic Alterations Associated with PTEN Mutation

**Figure 2:**
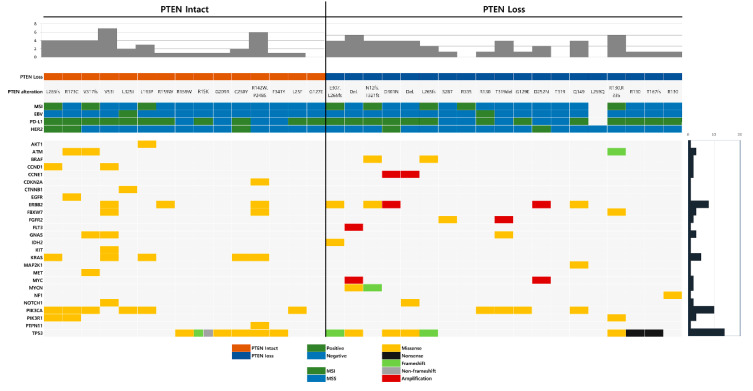
The distribution and prevalence of co-occurring genetic alterations associated with phosphatase and tensin homolog (*PTEN*), microsatellite instability (MSI) status, Epstein–Barr virus (EBV), HER2, and PD-L1.

Co-occurring genetic alterations with *PTEN* mutation were detected in 26 genes. The most common co-occurring genetic alteration was *TP53* mutation, found in 14 cases (41.2%), followed by *PIK3CA* mutation (10 cases; 29.4%), *ERBB2* mutation (8 cases; 23.5%), and *KRAS* mutation (5 cases; 14.7%). Translocation was not found in any of the cases. The distribution and prevalence of co-occurring genetic alterations associated with *PTEN*, MSI status, EBV, HER2, and PD-L1 are summarized in Figure 2.

## 3. Discussion

We performed comprehensive cancer panel tests in 322 patients with advanced GC and found *PTEN* mutations in 34 cases (10.6%), of which 19 (55.9%) showed PTEN protein loss. Among the cases with PTEN inactivation, nonsense mutation was the most common type, and all cases with nonsense mutation showed PTEN loss. *PTEN* mutations were significantly associated with PTEN protein loss, MSI-high, and EBV-positivity.

The frequency of *PTEN* mutation varies ranging from 0% to 20%, except for synonymous mutations [14,15,16,17,18]. Previous studies were mostly based on a small number of cases and included various disease stages. Further, the *PTEN* mutations were detected using Sanger sequencing or PCR-SSCP, which also included nonpathogenic mutations. In the present study, using NGS test (covering all hot and non-hot spot mutations) in cases with advanced GCs, the frequency of *PTEN* mutation observed was similar to the frequency (7–11%) previously reported in GCs [7,8].

Several mechanisms of PTEN inactivation have been suggested previously, including gene mutations, loss of heterozygosity, promoter hypermethylation, miRNA-mediated regulation, and post-translational phosphorylation [3]. In the present study, we focused on gene mutation and showed a significant association of *PTEN* mutation with the loss of its expression (*p* = 5.232 × 10^−10^). A previous study on *PTEN* mutations in GC used IHC in cases with PTEN nonsense mutations and reported an association between *PTEN* mutation and its expression. Further, the study showed that nonsense mutation reduced PTEN expression [15]. It has been suggested that *PTEN* gene inactivation, mainly due to mutation and/or loss of heterozygosity, plays a pivotal role in tumor progression. In TCGA GC data, all but one case with truncating mutations of *PTEN* showed decreased mRNA expression [7,22]. The present study confirmed that mutation, especially nonsense mutation, is one of the mechanisms of PTEN inactivation in GC. Given that a nonsense mutation results in a truncated and usually nonfunctional protein, we proved this change by directly comparing the type of *PTEN* mutation and protein expression in GC. 

We also found that *PTEN* mutation was significantly associated with MSI-high (*p* = 3.936 × 10^−8^), and EBV-positivity (*p* = 0.0071). Significant associations of PTEN mutation/inactivation and MSI-high were reported in colorectal cancers [23,24] and endometrial cancers [4]. The inactivation of PTEN has been reported in EBV-positive nasopharyngeal carcinoma through altered EBV-miR-BART1 expression [25] or promoter hypermethylation of *PTEN* in GC [26]. Several drugs are currently in clinical trials for the treatment of patients with PTEN-deficient cancers [27]. As EBV and MSI-high are predictive biomarkers for immunotherapy [28], either immunotherapy or combined therapy targeting PTEN would be a good option for this type of GC. A previous study investigated the association of DDR mutations (including *PTEN*) with MSI, PD-L1 expression, and tumor mutational burden (TMB) in GCs, and found that MSI-high, high PD-L1 expression, and TMB-high were significantly more frequent in DDR mutated cancers. In the study, *PTEN* was a prevalent DDR mutations found in MSI-H and TMB-high GCs (≥10 mt/MB) but not in GCs with high PD-L1 expression (CPS > 10) [29]. Another group reported that DDR mutated GCs had high TMB, but they did not assess the effect of PTEN on TMB [30]. As our study focused on *PTEN* alterations rather than DDR mutations, further studies are recommended to confirm the role and mechanism of failure of DNA repair in tumor immune dynamics in this era of cancer immunotherapy [31,32]. In previous studies, PTEN inactivation was significantly associated with tumor progression [3]; however, this difference was evident in the progression of early stage GC to advanced stage GC and the prognostic significance in patients with advanced GC is not evident [33,34]. In the present study with advanced stage GC, we also failed to find any significant correlations between PTEN inactivation and survival of the patients.

We analyzed co-occurring genetic alterations associated with *PTEN* mutations in GC and found alterations of *TP53* (41.2%), *PIK3CA* (29.4%), *ERBB2* (23.5%), and *KRAS* (14.7%) in all 34 *PTEN*-mutated cases. High frequency of coexistent mutations of *PIK3CA* and *PTEN* genes has been reported previously in endometrial carcinoma but not in GC [35]. These concomitant mutations can accelerate tumor progression through aberrant PI3K/AKT pathway. Although the relationship between *PTEN* and *ERBB2* has not been studied in GC, the frequency of PTEN inactivation in *ERBB2*-amplified GC has been reported in 16.4–34.5% of cases [36,37,38]. In the present study, five PTEN mutations (11.4%) and five PTEN protein losses (11.4%) were found in 44 GCs with HER2 overexpression. In HER2-positive GCs, PTEN inactivation has been linked to trastuzumab resistance [37,38,39]. The clinical significance of concurrent *KRAS* and *PTEN* mutations is unknown in humans but has been reported in murine carcinogenesis [40,41,42].

Overall, our NGS results showed *PTEN* mutation in 34 cases (10.6%) out of 322 patients with advanced GC. More than half of the GCs (55.9%) with *PTEN* mutation showed PTEN inactivation, of which all nonsense mutation showed PTEN inactivation. Although this was a retrospective study and the number of *PTEN*-mutated cases analyzed was limited, we demonstrated for the first time that *PTEN* mutation is significantly associated with PTEN protein loss, MSI-high, and EBV-positivity. Given that MSI-high and EBV-positive GCs are biomarkers for immunotherapy and the repertoire of PTEN functions has recently been expanded with new potential implications for immunotherapy-based approaches [43], further studies are warranted.

## 4. Materials and Methods

### 4.1. Patients and Data Collection

The study population consisted of a total of 322 patients with primary advanced gastric carcinoma registered at the Samsung Medical Center (Seoul, Republic of Korea) from January 2017 to July 2019. The study was approved by the institutional review board (IRB 2020-01-051). The median age of the study cohort was 61 years (29–85), and 203 (63.0%) patients were male. We obtained all tumor samples from the stomach (308 resections and 14 endoscopic biopsies). Clinicopathological information, including age, sex, sample type, and the method of sample collection were obtained from electronic medical records. All but two patients did not receive chemotherapy or radiotherapy prior to obtaining the specimen. Formalin-fixed paraffin-embedded (FFPE) tissues of specimens were used for several tests including NGS cancer panel test, MSI test, and immunohistochemistry (IHC) to find molecular characteristics of the GC. 

### 4.2. Cancer Panel Test with NGS

We performed NGS in all cases to detect copy number alterations (CNAs); single nucleotide variants (SNVs); small insertions and deletions (indels); and gene fusions using the Oncomine comprehensive cancer panel v1 (Thermo Fisher Scientific, Waltham, MA, USA), which examines 143 oncogenes and tumor suppressor genes. Our comprehensive cancer panel showed sensitivity of 99% for SNVs and 93% for indels at 10% allele frequency on the validation of analytical procedures using Acrometrix® Oncology Hotspot Control (Thermo Fisher Scientific) and 5-Fusion Multiplex FFPE RNA Reference Standard (Horizon Discovery, Waterbeach, UK). It also showed 100% sensitivity when the sample was diluted to 23% (CNAs) and 10% (fusions). For cancer panel tests, we used FFPE tissue samples, and the minimum tumor volume was 10%. After deparaffinization and nucleic acid extraction, targeted DNA and RNA amplification of the tumor was performed using the Ion AmpliSeq Library kit 2.0 (Thermo Fisher Scientific). Partial digestion of primer sequences, ligation of adapters to amplicons, and purification and quantitation of the libraries were performed using the Ion Xpress Barcode Adapter 1–96 kit (Thermo Fisher Scientific), Ion AmpliSeq Library Kit, and the Ion Library TaqMan Quantitation Kit (Thermo Fisher Scientific). Eight constructed libraries were loaded on an Ion 540 chip, and sequencing was performed using the Ion S5XL system. We used Ion Torrent software (Ion Reporter^TM^ 5.2, Thermo Fisher Scientific) and Oncomine Knowledgebase for automated data analysis. We set the following criteria and evaluated the sequencing quality accordingly: mapped reads > 5,000,000, on-target rate > 90%, mean depth > 1200, and uniformity > 90%. A sequencing coverage of 250X, variant coverage of 25X, and variant allelic frequency of 5% were set as cutoffs to avoid false-positive and false-negative results. An average copy number ≥ 4 was interpreted as a gain (amplification) and <1 as a loss (deletion). For translocations, read counts ≥ 20 and total valid mapped reads ≥ 50,000 were interpreted as positive results. 

### 4.3. Immunohistochemistry

To evaluate the correlation between genetic mutation and protein expression, we performed IHC with anti-PTEN antibody (138G6, CST, Danvers, MA, USA). Loss of nuclear and cytoplasmic staining in the tumor cells was considered as loss of PTEN expression. For HER2 IHC, we used anti-HER2 antibody (4B5, Ventana Mdical Systems, Tucson, AZ, USA), and scored HER2 status according to gastric criteria proposed by Ruschoff et al [44]. For EBV ISH, we used BOND-MAX with an EBV-encoded RNA probe (Leica, Newcastle, UK), and considered cases showing strong signals in most of the tumor cells as positive. For PD-L1 IHC, PD-L1 IHC 22C3 pharmDx kit (Agilent technologies, Carpinteria, CA, USA) was used with a Dako Autostainer Link 48 system (Agilent Technologies, Carpinteria, CA, USA). We measured the combined positive score (CPS) and the ratio of the number of all PD-L1 expressing cells to the number of viable tumor cells, as described previously [45]. 

### 4.4. MSI Test

MSI status was determined by multiplex PCR to amplify five quasimonomorphic mononucleotide repeat markers (BAT-25, BAT-26, NR-21, NR-24, and NR-27). Genomic DNA was isolated from the FFPE tissue blocks using a QIAamp DNA Mini Kit (Qiagen, Hilden, Germany). Sense primers were fluorescently end-labeled with FAM, HEX, or NED. Amplicons were analyzed on an ABI Prism 3130 Genetic Analyzer (Applied Biosystems, Foster City, CA, USA). Allelic sizes were estimated with GeneMapper 4.1 (Applied Biosystems, Foster City, CA, USA), and tumors with allelic size variation in <2 and ≥2 microsatellites were classified as microsatellite stable and instable, respectively.

### 4.5. Statistical Analysis

The χ^2^ test, Fisher’s Exact test, and Kruskal–Wallis rank sum test were used to compare categorical variables between *PTEN*-mutated and *PTEN* wild-type GCs. The Wilcoxon rank sum test was used to compare a continuous variable between *PTEN*-mutated and *PTEN* wild-type GC. The *p*-value was two-sided, and *p* < 0.05 was considered significant using R software version 3.4.4 (https://www.r-project.org/foundation/). 

## 5. Conclusions

In conclusion, PTEN loss-of-function mutation is an important genetic mechanism of PTEN inactivation.

## Figures and Tables

**Table 1:** Clinicopathologic characteristics of phosphatase and tensin homolog (*PTEN*)-mutated gastric cancers.

Case	Age	Sex	Specimen	Histology	HER2	EBV	MSI	PD-L1 CPS (≥1)
1	70	M	Stomach, biopsy	TADC, WD	-	-	MSS	-
2	58	M	Stomach, biopsy	TADC, MD	-	-	MSI	-
3	47	F	Stomach, biopsy	TADC, PD	-	-	MSS	+
4	65	M	Stomach, resection	TADC, PD	-	+	MSS	+
5	50	F	Stomach, resection	TADC, MD	-	-	MSS	-
6	53	F	Stomach, biopsy	TADC, PD	-	-	MSS	+
7	69	M	Stomach, biopsy	TADC, MD	-	-	MSS	-
8	61	M	Stomach, resection	TADC, PD	-	-	MSS	-
9	85	M	Stomach, biopsy	TADC, PD	-	-	MSS	+
10	69	M	Stomach, resection	TADC, MD	+	-	MSS	+
11	66	F	Stomach, resection	TADC, MD	+	-	MSS	-
12	41	M	Stomach, resection	TADC, PD	-	-	MSS	-
13	64	F	Stomach, resection	TADC, PD	-	-	MSS	-
14	64	M	Stomach, biopsy	TADC, PD	-	-	MSS	+
15	47	M	Stomach, biopsy	TADC, PD	-	NA	NA	NA
16	51	M	Stomach, biopsy	TADC, PD	-	-	MSS	-
17	56	F	Stomach, biopsy	TADC, PD	-	-	MSS	+
18	79	F	Stomach, resection	TADC, MD	-	-	MSI	+
19	77	M	Stomach, biopsy	TADC, PD	-	-	MSS	+
20	59	M	Stomach, biopsy	TADC, MD	-	-	MSS	+
21	66	F	Stomach, biopsy	TADC, PD	-	-	MSS	+
22	40	F	Stomach, biopsy	SRC	-	-	MSS	+
23	61	F	Stomach, resection	PADC, MD	+	-	MSI	+
24	78	M	Stomach, resection	TADC, PD	-	-	MSI	+
25	53	M	Stomach, resection	TADC, MD	+	-	MSS	+
26	76	F	Stomach, resection	TADC, MD	-	-	MSI	+
27	54	M	Stomach, resection	PADC, MD	-	-	MSS	+
28	79	M	Stomach, resection	TADC, MD	-	-	MSI	+
29	70	M	Stomach, resection	TADC, MD	-	-	MSS	+
30	60	M	Stomach, resection	TADC, PD	-	+	MSS	+
31	56	F	Stomach, resection	TADC, MD	-	-	MSI	+
32	76	M	Stomach, resection	TADC, MD	+	-	MSS	+
33	74	M	Stomach, resection	TADC, MD	-	-	MSS	+
34	76	M	Stomach, resection	TADC, MD	-	-	MSI	+

TADC = tubular adenocarcinoma; WD = well differentiation; MD = moderate differentiation; PD = poorly differentiation; SRC = signet ring cell carcinoma; PADC = papillary adenocarcinoma; MSS = microsatellite stable; MSI = microsatellite instability; CPS = combined positive score; NA = not applicable.

**Table 2:** Comparison of clinicopathologic characteristics between phosphatase and tensin homolog (*PTEN*)-mutated gastric carcinomas and *PTEN* wild-type gastric carcinomas.

	*PTEN*-Mutated (*n* = 34)	*PTEN* WT (*n* = 288)	*p*-Value
Sex			0.9805
Male	22 (64.7%)	181 (62.8%)	
Female	12 (35.3%)	107 (37.2%)	
**Age** (median, range)	64 (40–85)	61 (29–85)	0.175
**PTEN IHC**			**5.232 × 10^−10^**
Intact	15 (44.1%)	253 (87.8%)	
loss	19 (55.9%)	35 (12.2%)	
**MSI status**			**3.936 × 10^−8^**
MSI	8 (23.5%)	9 (3.1%)	
MSS	25 (73.5%)	279 (96.9%)	
NA	1 (3.0%)	0 (0%)	
**EBV**			**0.0071**
positive	2 (5.9%)	7 (2.4%)	
negative	31 (91.2%)	281 (97.6%)	
NA	1 (2.9%)	0 (0%)	
**HER2**			0.7945
positive	5 (14.7%)	39 (13.5%)	
negative	29 (85.3%)	249 (86.5%)	
**PD-L1 CPS**			0.111
≥1	24 (70.6%)	182 (63.2%)	
0	9 (26.5%)	105 (36.5%)	
NA	1 (2.9%)	1 (0.3%)	

WT = wild-type; MSI = microsatellite instability; CPS = combined positive score; NA = not applicable. Data with *p* < 0.05 are in bold.

## Supplementary Materials

The following are available online at https://www.mdpi.com/2072-6694/12/7/1724/s1, Table S1: Clinicopathologic characteristics of phosphatase and tensin homolog (*PTEN*) wild-type gastric carcinomas.
